# ‘I have built an office for myself in the sauna’ – The well-being of social workers in liminal space during the early phases of the COVID-19 pandemic

**DOI:** 10.1177/00208728231186519

**Published:** 2023-07-27

**Authors:** Sanna Saraniemi, Timo Harrikari, Vera Fiorentino, Marjo Romakkaniemi, Laura Tiitinen, Tuomas Leppiaho

**Affiliations:** University of Lapland, Finland; Finnish Institute for Health and Welfare (THL), Finland; University of Lapland, Finland; University of Lapland, Finland; University of Lapland, Finland; University of Lapland, Finland

**Keywords:** COVID-19 pandemic, everyday life, liminal space, remote work, social work, well-being

## Abstract

We examine the experiences of Finnish social workers regarding their well-being during the early phases of the COVID-19 pandemic by analysing social workers’ diaries (n = 33) using thematic analysis. The pandemic and the restrictive measures caused significant changes in work and private lives challenging social workers’ well-being. Restrictive measures provided a kind of liminal space where the boundary between work and private life, daily rhythms and relationships with coworkers and clients were redefined. This exceptional period required challenging demarcations to safeguard the well-being of social workers, but it also provided new options to promote their well-being.

## Introduction

At the beginning of 2020, COVID-19 spread around the world. In Finland, emergency conditions were declared on 16 March 2020, ushering extensive restrictive measures to prevent the spread of the virus. Although an absolute lockdown did not occur in Finland, many types of restrictions took place. For example, remote work was recommended, and several public services were downsized and offered services through remote access. Schools and universities moved quickly to remote teaching, and people were advised to avoid public meetings and physical contact with others (Harrikari et al., 2021; Salin et al., 2020). Overall, the pandemic and restriction measures brought a sudden change in people’s lives, both in terms of their work and personal lives.

The setting formed by the pandemic and associated restrictive measures can be characterised as liminal, a kind of intermediate space, where an individual is neither ‘in’ nor ‘out’ but ‘in-between’ (Turner, 1974; Van Gennep, 1960). Many structures that held both everyday life and working life together were questioned and restructured. During the early phases of the pandemic, a discussion about the ‘new normal’ emerged, but an overall uncertainty about the future prevailed, with no clarity regarding the length of the liminal space caused by the restriction measures.

Social services play a key role in supporting the most vulnerable people exposed to the negative effects of crises and in coping for society as a whole from crises and catastrophes (Banks et al., 2020). The restrictions caused a rapid and unforeseen change to social work organisations and work teams, as well as to the daily structures of employees and their clients. Social workers and social work organisations were forced to modify their working practices (Harrikari et al., 2021; Romakkaniemi et al., 2021). In addition, the pandemic increased the burden on social workers because they were also facing the impacts of the crisis on their own personal lives (Harrikari et al., 2021; McFadden et al., 2021). Therefore, it is essential to pay attention to the well-being and coping of social workers in a context where the conditions for implementing social work changed rapidly and social work began to be carried out in a liminal space. In analysing the effects of the pandemic on well-being at work, it is crucial to consider the links between work-related factors, personal factors and the wider societal context (Graham and Shier, 2010; Meyer et al., 2021; Orsila et al., 2011).

There are a growing number of studies concerning social work in the context of COVID-19. These studies have addressed topics such as the use of digital tools (Fiorentino et al., 2023; Mishna et al., 2021), human rights and social justice (Anand et al., 2022), ethics (Banks et al., 2020), social work education (Morley and Clarke, 2020), social workers’ resilience, mutual support and working communities (Cook et al., 2020; Saraniemi et al., 2022) and social workers’ well-being at work (McFadden et al., 2021).

In the current article, we examine the experiences of Finnish social workers during the early phases of the COVID-19 pandemic by using diary data collected between 16 March and 31 May 2020. We analyse the social workers’ descriptions about changes in their well-being in a situation where a multilevel crisis extended to both their working and private life spheres, forming a space of being ‘in-between’, where the two began to intersect. As our analytical framework, we apply the concept of liminality (Turner, 1974; Van Gennep, 1960) and the multidimensional conceptualisation of well-being at work (Orsila et al., 2011). The diaries were analysed using thematic analysis, highlighting the three key themes related to social workers’ well-being in the early stages of the pandemic: the breakdown of everyday routines, questioning the temporal and spatial solutions of everyday life and the emergence of new and alternative supportive rhythms and practices.

## Changes in work in the liminal space

The concept of liminality refers to being in an intermediary space or threshold as a kind of transitional phase. It has been defined as a nonlinear threefold process or structure. A classic scholar in this area, Arnold Van Gennep, states that in the first phase, there is separation from the known and familiar – separation from the everyday life practices, places and relationships. The second stage is the liminal space, a kind of waiting room or threshold where the normal roles, structures and norms cannot be applied anymore. Finally, in the third phase, there is the formation of a ‘new normal’ (Van Gennep, 1960).

The liminal space has been described as a state of uncertainty and discomfort that can provoke anxiety as the previous order, predictability of events and the sense of security have been broken: within this new space, there is no certainty on how things will be organised in the future (Douglas, 1966; Van Gennep, 1960). However, the liminal space can also be seen as a state of open possibilities and hope, allowing for the transition from the old structures towards something new (Turner, 1974). As everyday routines become questioned, occasions for reflecting on values can emerge. Besides shifting towards something new, liminality can also end on its negation – reinstatement of the old order and structures without implementation of the lessons learned during the tribulating liminal phase (Turner, 1974; Van Gennep, 1960). Individuals may experience the liminal space differently, which is affected by, among other things, one’s personal life situation and work history (Wilson et al., 2021).

In the context of work, this may refer to, for example, working in different spaces and times, reflecting critically on past routines and rhythms and rhythmising work differently, which can affect well-being at work. In studies on working life, the concept of liminality has been used, for example, to study alternative working environments (Vesala and Tuomivaara, 2019) and in describing the overall character of current working life (Izak et al., 2023). Liminality has also been applied, for example, in studies of the well-being of health care workers during the COVID-19 pandemic (Cook et al., 2021). Previous studies highlight how liminality causes burden and uncertainty, but it can also offer new opportunities when, for example, ordinary working rhythms are questioned.

We understand the setting formed by the pandemic and restrictive measures as a ‘liminal space’. The onset of the COVID-19 pandemic caused a rapid change in working life as employees had to avoid physical encounters and switch to a remote working mode. Although continuous changes and multilocational work have been a growing trend in working life (Felstead and Henseke, 2017; Korunka et al., 2015), the experience of working remotely before the pandemic was limited. There were no ready-made guidelines for working remotely, and impromptu rules had to be made in an unprepared and forced manner (Wang et al., 2021). In Finland, remote working was not well established in social work before the pandemic (Harrikari et al., 2021), forcing social workers to adapt quickly, not only to their working practices, but also to their private lives, forming a space of being ‘in-between’. The private sphere began to intersect with the professional work sphere. Many structures that upheld the balance between work and everyday life were brought into question and had to be restructured and rethought. During the early phases of the pandemic, a discussion about the ‘new normal’ emerged, but an overall uncertainty about the future and duration of the restrictive measures prevailed (Harrikari et al., 2021).

In the context of social work, liminality has been used to describe the nature of social work and to analyse the life situations of social work clients and their identity processes (Warner and Gabe, 2004; Wilson, 2019). Liminality has been used, for example, in the contexts of social work research to examine how the pandemic has affected perinatal social work (Wilson et al., 2021) and vulnerable populations, such as disadvantaged youths (Woodrow and Moore, 2021).

## Social work in the liminal space and the challenges to professional well-being

Well-being at work is a multidimensional phenomenon. It is affected by organisational factors, along with those factors related to the working community, the relationship between the demands of work and the resources of the employee and employees’ personal, physical, psychological and social well-being. For example, the multidimensional perspective highlights how organisational factors like management and flexibility, intrinsic factors like personality and workability but also other aspects of life, such as family situation and wider societal context, affect well-being at work, not to mention how the different dimensions interact with each other (Graham and Shier, 2010; Meyer et al., 2021; Orsila et al., 2011).

Multilocational work raises new types of challenges and opportunities regarding the issues of well-being at work. On the one hand, work detached from time and place allows employees greater flexibility to plan their schedule, contributing to a better balance between work and private life (e.g. Gajendran and Harrison, 2007). On the other hand, the challenges of well-being in remote work consist of (in)adequate ergonomics, the employee’s loneliness, alienation from the work community, separating work and leisure and recovering from work. It can be difficult to separate well-being at work from personal, out-of-work well-being. The role of the closest superiors in supporting workers’ well-being is significant, but in multilocational work, well-being remains mainly the employee’s own responsibility (Felstead and Henseke, 2017; Wang et al., 2021). Therefore, maintaining well-being in a changing working life requires employees’ resilience and adaptive capacity, flexibility or the ability to adapt and recover from rapid changes and develop new ways of working (Youssef and Luthans, 2007). Meyer et al. (2021) highlight the links between work-related factors, personal factors and the broader societal context, such as the impacts of restrictive measures imposed by the government, when analysing well-being at work in the crisis context.

Social workers are known to be motivated and committed to their work, but they are often heavily overloaded. Staff shortages, financial pressures and requirements of efficiency tend to weaken the well-being of social workers and their opportunities to perform professionally sustainable work (Mänttäri-Van der Kuip, 2014). The pandemic caused mental challenges for social and health care workers, such as anxiety and insomnia related to fear of infection, a lack of protective equipment and inadequate or unclear guidelines for protection. Front-line workers were concerned about carrying the virus home and infecting loved ones (Sheraton et al., 2020). Ethical challenges related to the implementation of social work during pandemics have also increased the burden on social workers (Banks et al., 2020). This concern was also compounded by the fact that there was no precise information on the duration of the pandemic or on the nature of the disease. It was also unclear when the situation would return to normal.

The burden of balancing work and family life during the pandemic is a key issue that has been highlighted in numerous studies. When employees with families shifted to remote working mode, other family members shifted to distance schooling or worked through remote connections. During the pandemic, women have had the main responsibility for housework and have had to reconcile their roles as mothers and workers, in turn increasing their mental strain (Meyer et al., 2021; Sharma and Vaish, 2020). In addition to experiences of loneliness and isolation, the mixing of work and private life, lack of interaction, difficulties in starting work days and increased workload have likewise been regarded as major challenges in remote working. In coping with this situation, not only has the importance of support and guidance from superiors been emphasised, but also flexibility in reconciling work and family lives has been deemed important (Meyer et al., 2021; Wang et al., 2021). In addition, the importance of continued learning and self-directed personal positive coping strategies has been emphasised (McFadden et al., 2021).

## Methodology: Data, ethics and method

### Study design and data collection

The empirical data consist of social workers’ diaries (*N* = 33) compiled between 16 March and 31 May 2020. The request for diaries was presented on a closed Facebook group for social workers that had more than 4000 members at the time of data collection. The prompt for the participant requested the participants to write diaries based on the following questions: (1) ‘What kinds of observations and experiences do you have about the phenomena and challenges that occur in the lives of social work clients during the pandemic?’; (2) ‘What challenges do social work and its practices face during a pandemic?’; and (3) ‘What kind of thoughts does the pandemic period evoke in you as a social work professional?’ The respondents were instructed to write freely but to date their diary entries. The research team received 33 entries from social workers (aged 30–53 years), with a few social work students among the participants. The participants worked in different areas of social work: in child protection, health care, social work with adults, elderly people, disabled people, immigrants and with people with addictions. Most wrote entries on a day-by-day basis, some week-by-week. In total, 94,139 words were collected.

In terms of ethical considerations, the research team followed the guidelines of the Finnish National Board on Research Integrity (TENK, 2019). The participants were informed that writing a diary was voluntary and sending it to the research team meant giving one’s consent. The background information gathered was limited: age, gender, professional title and education, current working title, and the main client groups with whom the participant was currently working. Institutional background information was not collected. All identifiable personal information was anonymised.

### Data analysis

The diaries were analysed using thematic analysis, employing both data-driven and theory-driven elements (Braun and Clarke, 2006). After the initial reading phase, all descriptions related to the social workers’ well-being were highlighted and coded. Next, the descriptions were organised into potential themes reflecting on the conceptual framework. At this stage, the diaries were searched for descriptions in which the social workers described those factors that challenged their well-being during the liminal state and for descriptions of factors supporting well-being and coping within the liminal state. As analysis of the data highlights, issues affecting the well-being of social workers blurred when the lines between work and private life began to fade and change because of the restrictions, and for this reason, we do not distinguish between well-being at work and personal well-being as different categories. As the main factors influencing the well-being of social workers in the liminal state caused by the restrictions, three themes emerged: (1) the breakdown of everyday routines: everyday structures were rapidly called into question and unpredictably greatly increased because of the restrictions and fear of contagion; (2) liminal spaces and places: the boundaries between work and private spheres supporting social workers’ well-being began to fade and blur; and (3) spatial and temporal solutions: new and alternative supportive rhythms and practices to replace the structures and practices emerged. These findings are presented in accordance with the phases of the liminal process (Van Gennep, 1960).

## The collapsing structures of everyday life

The first experiences of social workers in the early period of the liminal stage meant a separation from the known and familiar, and from everyday life practices, places and relationships (Van Gennep, 1960). The pandemic setting was an unprecedented one: the restrictive measures permeated people’s everyday rhythms, temporal and spatial structures of everyday lives and social relations, both in their private and working lives. In the beginning, social workers described their state of mind by using terms such as ‘shock’ and ‘unreal’ (also Sheraton et al., 2020) because they were suddenly finding themselves in a situation where the old norms, structures and practices no longer worked. The pandemic was omnipresent, with the media being filled with news about it. Their everyday ‘choreography’ had to be restructured to minimise physical contact. In social work, where face-to-face interaction was once at the centre and where remote working was rare, the avoidance of physical contact required fundamental changes to one’s work practices:

Work has suddenly become quite different because the encounters between colleagues and clients are different. This leads to confusion about how a job can be done. Since learning, reflecting and reasoning take time. How do I promote client involvement when I can’t face them? (D22/20/03/26)

The requirement to limit physical contact and the extensive shutting off of Finnish society raised concerns among social workers about the well-being and coping of clients and the implementation of ethical principles in their social work. Several supportive services were closed and communicated remotely with their clients. New working methods had to be found to navigate forward during the crisis. Although several public services were closed, social work had to continue:

Social work cannot take a break, even if other functions of society slow down. An exceptional situation does not eliminate existing problems; the biggest ones remain, and we should be able to grip them despite the epidemic. (D14/20/03/20)

There were hardly any ready-made instructions for restructuring work practices in this rapidly changing situation, and the experiences of liminality were strongly present within the diaries. The social workers described how the instructions related to meeting clients could change even daily. The decisions and demarcations regarding which of the clients must be met face to face and how to ensure data security while working outside the office or how to prevent the spread of infection in face-to-face encounters with clients remained the employees’ responsibility.

In their diaries, the social workers described the burden, stress and ethical challenges posed by unclear and incomplete guidelines (also Banks et al., 2020). IT equipment, such as microphones, laptops or software, was not available, especially in the early stages of the pandemic. In many respects, the transition to remote work was unplanned, and new practices had to be developed quickly. Indeed, the initial stage of the pandemic appeared mainly as *confusing* and *chaotic* (Douglas, 1966; Van Gennep, 1960).

Moreover, the restrictive measures affected the social workers’ private lives. Those with families, especially women (Meyer et al., 2021; Sharma and Vaish, 2020), faced ‘role conflicts’ as spouses and children switched to remote work and distance learning. The family’s food supply and practical organisation of school days caused contradictions, leading to time pressures:

This Friday ends with the supervision of my daughter’s math exam at home. Everyday life has changed, both at home and at work, everywhere! I find myself more tired and busy this week than I used to be: all sorts of new ways of working (including meeting clients with masks on their faces), and the constant discussion about the corona burdens me. As I am not only an employee, but also a spouse, mother, friend and daughter of my parents who are already over 70 years old. (D30/week 13)

The social workers were concerned about contagion. There were several concerns related to the illness of both oneself and one’s own family members, accompanied by the fact that one could unknowingly infect clients or coworkers. This caused mental stress for the social workers at the interface between work and private life. One of the social workers wrote, ‘*I woke up at night to consider work and the safety of my children*’ (D10, week 13). There were also fears about one’s own situation:

Because I have lung disease, I am in the corona risk group, and on Monday, I was terrified of going to work. . . . I hid myself all day, working alone in my office behind a locked door. I scheduled my breaks so that there were no others there in the breakroom. I heard some coworkers’ laughter through the door. One coworker sent a message in Teams that she was alone in her room crying because the situation was so distressing and frightening. (D16/20/03/24)

The sudden collapse of the structures of everyday lives caused liminality within social workers’ lives. The beginning of the liminal state turned out to be a confusing and stressful period in which social relations quickly changed both at home and at work. The normal structures of everyday life had faded, with no certainty on how life would be organised in the future (Douglas, 1966; Van Gennep, 1960).

## Liminal spaces and places

In liminal space, the normal structures and norms cannot be applied anymore (Douglas, 1966; Van Gennep, 1960). The demand to limit face-to-face encounters quickly shaped the use of the everyday spaces of social work in a fundamental way. With the overall mandate to work remotely, some social workers moved to work from home. They described how work used to be clearly demarcated outside their homes and private lives but how the boundary between work and home now began to blur. Those with families had to reconcile their own work with the rhythms of other people at home:

There is sometimes quite a mess at home because there is someone in every corner holding a meeting, etc. (D2/20/04/15)

The home, which had previously been a place of privacy and recovery from work, had to be transformed into a working space. This required organising spaces at home, making arrangements that made working remotely possible and meeting clients through remote access. Issues related to client confidentiality, data security and ergonomics remained the social workers’ own responsibility. Consequently, the boundaries between work and private life had to be redefined by creating spatial solutions that enabled taking time off from work:

I have built an office for myself in the sauna. The stand is ergonomic; it is a laundry basket on top of benches. Adjusting the height is possible with the help of a washing pan. (D6/20/03/24)

While working from home, the physical distance from colleagues increased. Small but supportive everyday encounters between coworkers were left out of the working day. While working remotely, employees had to call a coworker, which meant the threshold to ‘bother’ a coworker increased. The social workers described how the transition to remote work caused both loneliness and isolation. However, continuing to work physically in the workplace did not necessarily remove feelings of loneliness because many coworkers worked remotely, and face-to-face encounters were minimised through various arrangements. Some social workers described how matters of work could stay on their minds afterwards because it was not possible to debrief with coworkers during the work days (Sharma and Vaish, 2020; Wang et al., 2021). In addition, because of the sudden shift to remote working and the widespread closure of society, there were no alternative means to gain distance from work:

Fatigue with everything, and the corona adds to it. The days follow one another; work and leisure are one and the same. Working feels heavy . . . All the shit that flows right from work to home now somehow remains here. I do not feel like wading in it from day to day. (D18/20/04/26)

Fear of contagion and the lack of necessary protective equipment led to the avoidance of face-to-face encounters altogether, incessant monitoring of one’s own health and symptoms and painful ethical discretion and demarcation on whether the benefits of facial encounters would outweigh the disadvantages of possibly becoming infected (also Banks et al., 2020). Avoidance manifested concretely in interactions between coworkers, which, according to several diary authors, was a key prerequisite for coping at work and is an essential element of their well-being at work (Shier and Graham, 2011). Although some social workers continued to work physically in the workplace, the nature of collegial encounters changed because fear of infection affected the atmosphere:

Most people are in shock. Some people who work remotely already miss being back at work with others; some of them I don’t hear anything from. There are still some people present at the workplace . . . Some of them wash their hands always before touching any new surface . . . No one sits close to each other anymore, and the coffee room is not filled with happy conversation during meal breaks. No one goes to pick up lunch from a store, but everyone eats snacks made at home. Faces are serious; the atmosphere is quiet. (D9/20/03/23–29)

This liminal state had many impacts on social workers’ well-being. The shift to remote work, claims to keep physical distance and the fear of viral infection related to physical encounters, changed the relationship between colleagues, weakened collegial support and caused feelings of social distance and loneliness. When working remotely from home, different roles and responsibilities, such as the roles of employee and parent, started to mix, causing extra burdens and confusion (Van Gennep, 1960).

## Changes in temporal rhythms in the liminal space

In the liminal space caused by restrictive measures, the former temporal structures decomposed, and there was no longer certainty how things would be organised in the future (Douglas, 1966; Van Gennep, 1960). Our diary data are teeming with various temporal attributes, showing the uncertainty regarding the duration of the pandemic and restrictions and reflections on when they would be able to return to normal everyday life. Exceptional conditions confused the social workers’ rhythms of everyday life, work and leisure. The social workers described how the days of the week did not differ and how ‘time’ had lost its significance (Wilson et al., 2021). Regular rhythms, repetitive routines and transitions between places and breaks, which usually helped control the use of time and limit the time spent at work, faded away with the restrictions. The social workers described how similar days followed each other and how work began to stretch into the evenings:

The past four weeks have been the same suffering. Weekdays and weekends blend smoothly into the same mess. Every evening, you must iterate what day it is, what time you have to set on the alarm clock and how busy you will be in terms of setting yourself in front of the screen. (D18/20/04/10)

The workers’ diaries described the importance of daily routines and regular rhythms for coping and well-being: everyday life ‘keeps rolling’ when it is predictable and repeats its safe formula. However, with the first wave of the pandemic, the familiar time–space structure of everyday life was questioned, rhythms changed, and predicting the future became difficult. Adopting new routines requires extra effort from employees. In addition, holiday periods, which were used to create a rhythm in everyday life, were shrouded in the uncertainty caused by the duration of the pandemic.

However, the entries in the diaries on changes in rhythms are twofold. On the one hand, moods are described as waiting and stopped, and some diary entries imply a slowdown in everyday life brought about by remote working. However, the liminal state also appears as a state of open possibilities that allowed for the transition from the old structure towards something new (Turner, 1974). As the weeks went by, the social workers also started to critically reflect on their former working routines and practices from the perspective of well-being at work. While working remotely from home, it was possible, at least to some extent, to create a rhythm and pause the workday in ways that supported one’s own personal coping and well-being the best.

Now, however, I’ve found that I cope better when I work partly at home. I still believe that the main contributors to endurance are microbreaks. In the workplace, work is done intensively all day, and even if you work all the time at home, you can sometimes hug your dog or go out and turn your seedlings to a different position from the sun. You’re not so nervous about being completely exhausted after workdays. It feels like you can work at home at a pace suitable for yourself. (D16/20/04/15)

On the other hand, there were descriptions indicating that the work rhythm remained busy or even accelerated. During the pandemic, the workload was not evenly distributed between the different sections of social work, but some sections, such as child protection and residential care, were subjected to more urgency and time pressure than others. The descriptions of the accelerating work rhythms were related to, among other things, the performance of ‘corona tasks’ assigned along with the typically heavy workload. Through remote connections, it was also possible to hold meetings on a tighter schedule because moving from one place to another did not take any additional time (Korunka et al., 2015). Moreover, with remote working, coffee and lunch breaks in the work community no longer had the rhythm of typical working days. One of the social workers described this situation as ‘*being bored remotely*’.

Indeed, several diaries considered whether work in social services could be multilocational in the future so that these services could be performed flexibly at home, at the office or elsewhere. The well-being and coping of employees would benefit from self-organised rhythms and the flexibility that multilocationality brings to their work. Thus, the liminal state posed by restrictions also opened up possibilities for critical reflection on former working practices and routines, providing ways to construct new rhythms and routines that could be the elements of the possible ‘new normal’ (Turner, 1974; Van Gennep, 1960).

## Discussion

In the present article, we have analysed the experiences of Finnish social workers regarding their well-being at work during the early phases of the COVID-19 pandemic. Initially, the pandemic and restrictive measures created a liminal state in people’s daily lives, one that was marked by uncertainty about the duration of the state of emergency; there existed an uncertain space between the old and new (Van Gennep, 1960). Although liminal space intersected with people’s everyday lives and society, it affected people in different life situations in different ways. There were entries among the diaries that life had continued in a relatively normal way. As a watershed moment, the effects of the restrictions on the lives of families and single people were evident. Thus, the diaries highlight how employees’ well-being was influenced by personal, organisational and societal factors and how these different dimensions intertwined with each other (Meyer et al., 2021). Many work practices, everyday routines and familiar rhythms that supported social workers’ well-being quickly disappeared, but there were no established practices to replace them. All of this resulted in social workers being forced to reflectively and individually restructure their daily lives within this liminal space.

The diaries were analysed using thematic analysis highlighting the three key themes related to social workers’ well-being in the early stages of the pandemic: *the breakdown of everyday routines, questioning the temporal and spatial solutions of everyday life* and *the emergence of new and alternative supportive rhythms and practices.* Although the sudden changes in work practices and everyday life in the early stages of the pandemic were described as stressful and challenging (Meyer et al., 2021; Sharma and Vaish, 2020; Sheraton et al., 2020), as the weeks passed, the social workers began to reflect on the potential of digitalisation and multilocational work as factors that could also support their well-being (Harrikari et al., 2021; McFadden et al., 2021). The state of emergency forced the social workers to see opportunities that could be utilised in social work in the future as factors that could increase the employees’ job satisfaction and well-being as well as reduce their workload. Working remotely provided opportunities to structure the working day according to one’s own rhythm and focus on work tasks without interruption. Indeed, the liminal space provided an opportunity for critical reflection on work practices because many everyday structures were questioned and became the subject of scrutiny.

Life in the liminal space made visible the key temporal and spatial dimensions structuring everyday life, implicitly affecting the well-being of social workers. These dimensions include the regulation of work–leisure interfaces (Salin et al., 2020) and the everyday rhythms and relationships with coworkers (Cook et al., 2020). The transition to remote working and the changed work practices required a redefinition of the boundary between work and private life to ensure personal coping (McFadden et al., 2021). The results suggest that organisational flexibility and employee autonomy to determine the amount of remote work and draw the line between distance and office days in compliance with one’s own resilience could contribute to well-being at work (Wang et al., 2021). However, the conclusion is not unambiguous; rather, the effect of remote work on well-being at work is linked to many other factors, such as available collegial support, the employee’s career stage and the employee’s family situation (Cook et al., 2020). With the fear of viral infection and prevention of infections necessitating the limiting of face-to-face encounters between colleagues and moving to telecommuting, the experience of collegial support weakened.

Based on the results, we would like to make some recommendations for practice to support social workers’ well-being during exceptional circumstances. First, it is important that organisations and superiors recognise the varying life situations of their workers – including family situation, health and living arrangements – to offer adequate support required to reconcile between work and private life. This type of support could take many forms, such as offering guidance on organising working rhythms and routines, and by allowing flexibility. Organisations ought to find concrete ways to support and cultivate individual adaptive capacities. Furthermore, facilitating dialogue not only between superiors and workers but also among workers themselves would be important for easing the feelings of loneliness and allowing a dialogical space for debriefing burdensome situations. During remote work, possibilities for debriefing have to be ensured adequately (Saraniemi et al., 2022). Providing the necessary IT equipment and applications for remote work would be essential as well as providing instructions for their use in both an effective and safe manner (Harrikari et al., 2021). In pandemic contexts, it would be important to provide the necessary equipment to protect the worker from contagion, too. Finally, establishing both ethical and practical guidelines for remote social work would ease the dilemmas pertaining to ethical conduct and privacy concerns.

## Limitations

The present study was implemented in the Finnish context and with qualitative methods, which should be kept in mind when interpreting the findings. Qualitative diary data can provide an authentic but limited view of everyday social work practices. The authors of the diaries were selected from workers for whom writing is a natural way to reflect on their thoughts and experiences. The experiences of social workers and the episodes they have described in their diaries are also mediated by their very nature, focusing only on the first wave of the pandemic.

## Conclusion

After three years, much has been learned about COVID-19, its properties and how to deal with it. Considering the lessons learned, the first wave and its implications for social work must be related to the procedural framework of the pandemic: there was a shock phase of the crisis, in which people’s everyday and organisational routines had to be adapted to what was unknown at the time. This is also emphatically apparent in our unique dataset that was gathered within this exceptional period of time. In terms of confronting and coping with possible forthcoming crises, our analysis highlights the importance of maintaining and strengthening social relations and networks at the workplace in times of crisis. It is crucial to ensure the availability of collegial support in these situations. In addition, as a practical implication, our findings highlight the importance of preparing organisations and working communities to confront crises. It seems that the employees confronted the crisis mainly individually, which amounted to an increase in stress and overall burden. In general, employees need concrete support to reconcile work and private life and different roles and responsibilities during exceptional circumstances. The results show how social work organisations should be more closely involved in the future in preparing for, preventing and responding to an increasingly unstable and unpredictable global environment (Rapeli, 2018). It is also important to promote the best practices identified during the pandemic that can support the resilience and well-being of social workers. The well-being of social workers must be taken care of as a condition for contingency planning and preparedness for future social work.
